# Editorial: Therapeutic drug monitoring and clinical toxicology of anti-cancer drugs, volume II

**DOI:** 10.3389/fonc.2023.1153714

**Published:** 2023-03-07

**Authors:** Miao Yan, Yao Liu, Jennifer H. Martin

**Affiliations:** ^1^ Department of Pharmacy, the Second Xiangya Hospital of Central South University, Changsha, Hunan, China; ^2^ Department of Pharmacy, Daping Hospital of Army Medical University, Chongqing, China; ^3^ School of Medicine and Public Health, University of Newcastle, Newcastle, NSW, Australia

**Keywords:** therapeutic drug monitoring, clinical toxicology, anti-cancer drugs, editorial, precision medicine

This is the second editorial in this series examining new oncology therapeutics and applications for which therapeutic drug monitoring (TDM)– adjusting dosages to improve outcomes to individualise patient care – can help individualise drug dosage. In general, TDM has been known to be helpful for over 50 years in most of the chemotherapy dosing of the older but still mainstream oncology therapeutics e.g. the taxanes, 5FU, methotrexate, etc. It has also shown significant utility with TKIs as discussed in the first series as these drugs also have a narrow therapeutic window, display significant inter- and intra-individual pharmacokinetic variability, and have a known concentration-effect relationship which can be utilised by the treating doctor. TDM is now finding additional uses as new drugs are being developed, and because patient physiology is increasingly variable compared to the previous generation. Older and more medically vulnerable patients are also now able to tolerate such chemotherapies now - due to the fact inter alia that supportive therapies e.g. ICU and transplant technologies are available to patients who previously would not have been offered chemotherapy due to comorbidity e.g. reduced organ function, age or concomitant medication. The other new development in this area is the development of an increasing number of oral agents and their combinations, including different families of tyrosine kinase inhibitors, many of which have significant activity in the P450 system and are often substrates for this system also, leading to widely variable dose-exposure relationships.

Stojavana et al. stated in a short report using a rapid snapshot of the literature, new biologics are also entering clinical practice, some based on minimally measured patient pharmacokinetic data, many of which are marketed at a single, maximally tolerated fixed dose, the same dose for each patient regardless of their physiology, comorbidity, diet, and concomitant medications. They note that for the most part, research initiatives are academic, and the evidence base has unfolded according to the clinical need and specialist areas of research groups. This report and the in-depth review of TDM of three commonly used chemotherapies (busulfan, 5-FU, and methotrexate) by Smita et al. in this context did set the scene for 10 other research reports.

Lin et al. used a SEER population-based analysis and examined the risk of heart disease-related death (HDRD) among anaplastic astrocytoma patients after chemotherapy. In this registry, 7129 anaplastic astrocytoma patients were studied, counterintuitively showing that those treated with chemotherapy compared to those not treated with chemotherapy were associated with a lower risk of HDRD. However, it is noted that the data are registry-based and thus open to large confounding. One likely confounder is that older and more frail patients are not offered some or any chemotherapy due to the risk-benefit ratio being higher than the less frail and younger cohort. And as HDRD is age-related, this could explain such a finding.

Yu et al. used data from 149 breast cancer patients receiving lapatinib to predict a personalised dose regimen using twelve machine learning and deep learning techniques. They chose TabNet to construct the prediction model with the best performance and then ranked four variables that strongly correlated with lapatinib dose: treatment protocols, weight, number of chemotherapy treatments, and number of metastases. Finally, the confusion matrix was used to validate the model for a dosing regimen of 1,250 mg lapatinib (precision = 81% and recall = 95%) and a dosing regimen of 1,000 mg lapatinib (precision = 87% and recall = 64%). A confusion matrix (also known as an error matrix) is a specific table layout that allows visualization of the performance of an algorithm in machine learning. However, although this model shows good predictive performance in a retrospective audit, validation in a new population and comparison to existing algorithms have not been undertaken.

Tan et al. studied genetic polymorphisms in CYP2C19 in 139 Chinese patients with lung cancer and studied the effect of these on exposure and adverse events of anlotinib, a small molecular multi-targeting tyrosine kinase inhibitor (TKI). As seen with other TKIs, there were significant variances (nearly 20-fold) in plasma trough concentration (3.95–52.88 ng/ml) and peak plasma concentration (11.53–42.8 ng/ml) following administration of 8 mg anlotinib; similar variances were seen with the 12mg dose tablet. Specific genetic mutations in CYP 2C19 accounted for much of this. Importantly, the mutations in CYP2C19 and corresponding higher exposures were correlated with higher incidences of hypertension and hemoptysis.

TDM requires timely access to a validated drug measurement system. This can be expensive when only a few patients are using a wide variety of oncology drugs. In order to make this more efficient, Jiang et al. developed a rapid determination of nine Tyrosine Kinase Inhibitors for the treatment of hepatocellular carcinoma in human plasma by QuEChERS-UPLC-MS/MS using the QuEChERS (Quick, Easy, Cheap, Effective, Rugged, and Safe) method. Lenvatinib, sorafenib, cabozantinib, apatinib, gefitinib, regorafenib, and anlotinib showed reasonable linearity over the range of 0.1–10 ng/ml, with the range 1–100 ng/ml showing linearity for tivantinib and galunisertib. All the linear correlation coefficients for all standard curves were ≥ 0.9966. The limits of detection and the limits of quantitation range were reasonable. The method was deemed satisfactory with an accuracy of -7.34–6.64%, selectivity, matrix effect (ME) of 90.48–107.77%, recovery, and stability. The proposed method is simple, efficient, reliable, and applicable for the detection of multiple commonly used TKIs in human plasma samples.

Lenalidomide (LEN) therapy is important in multiple myeloma (MM) and non-Hodgkin lymphoma (NHL), but requires dose adjustment in renal impairment. However, the optimal concentration range has not been clearly defined. Song et al. undertook a prospective observational study of the exposure-safety relationship of LEN to determine the target concentration for toxicity. Out of the 61 patients enrolled in this study, hematological toxicity was reported in 15 (24.59%) patients. The LEN C_min_ showed remarkable differences (*p =* 0.031) among patients with or without hematological toxicity, while no association between C_1h_ values and toxicity was noted. By ROC analysis, a C_min_ threshold of 10.95 ng/mL was associated with the best sensitivity and specificity for toxicity events (AUC = 0.687; sensitivity = 0.40; specificity = 0.935). By multivariate logistic regression, a LEN C_min_ below 10.95 ng/mL was associated with a markedly decreased risk of hematological toxicity (<10.95 ng/mL vs. >10.95 ng/mL: OR = 0.023, 95% CI = 0.002–0.269; *p* = 0.003).

Chen et al. sought to understand immune-related adverse events in NSCLC patients with concomitant hypertension in patients receiving PD-1/PD-L1 inhibitors using disproportionality analysis in the Food and Drug Administration (FDA) Adverse Event Reporting System (FAERS) database. Among 17,163 NSCLC patients under treatment with a single-agent anti-programmed death-1/programmed death ligand-1 (PD-1/PD-L1) inhibitor (nivolumab, pembrolizumab, cemiplimab, durvalumab, atezolizumab, and avelumab), 497 patients had hypertension, while 16,666 patients had no hypertension. 4,283 pulmonary AEs were reported, including 166 patients with hypertension and 4,117 patients without hypertension. Compared with patients without hypertension, patients with hypertension were positively associated with increased reporting of interstitial lung disease (ROR = 3.62, 95% CI 2.68–4.89, IC = 1.54, IC_025_ = 0.57) among patients receiving anti-PD-1 treatment.

Cardiotoxicity is a well-known pathophysiological consequence in breast cancer patients receiving trastuzumab. Trastuzumab-related cardiotoxicity typically results in an overall decline in cardiac function, primarily characterized by a reduction in left ventricular ejection fraction (LVEF) and the development of symptoms associated with heart failure. Current strategies for the monitoring of cardiac function during trastuzumab therapy include serial echocardiography, which is cost-ineffective as well as offers limited specificity, while offering limited potential in monitoring early onset of cardiotoxicity. However, biomarkers have been shown to be aberrant prior to any detectable functional or clinical deficit in cardiac function. Pillai et al. aims to develop a panel of novel biomarkers and circulating miRNAs for the early screening of trastuzumab-induced cardiotoxicity. Patients with a clinical diagnosis of invasive ductal carcinoma were enrolled in the study, with blood specimens collected and echocardiography performed prior to trastuzumab therapy initiation at baseline and 3, 6 months after trastuzumab therapy, respectively. Following 6-months of trastuzumab therapy, about 18% of the subjects developed cardiotoxicity, as defined by a reduction in LVEF. The results showed significant upregulation of biomarkers and circulating miRNAs, specific to cardiac injury and remodeling, at 3- and 6-months post-trastuzumab therapy. These biomarkers and circulating miRNAs significantly correlated with the cardiac injury-specific markers, troponin I and T. The findings in this study demonstrate the translational applicability of the proposed biomarker panel in the early preclinical diagnosis of trastuzumab-induced cardiotoxicity, further allowing management of cardiac function decline and improving health outcomes for breast cancer patients.

de Vries et al. have investigated whether high exposure is a reason for the discontinuation of pembrolizumab due to immune-related adverse effects (irAEs). This is important as these drugs are very effective and discontinuing them may aggravate the disease of patients. They note the currently available pharmacokinetic (PK) and pharmacodynamic (PD) data to reassess these dosing strategies are insufficient and based on data that is not directly relevant to clinical practice. They highlight the importance of TDM by using plasma measurements after a single 200 mg pembrolizumab dose in a treatment-naive patient with non-small cell lung cancer (NSCLC). Their work notes the complexity of drug exposure, receptor occupancy, and the T-cell effect, and how simple PK-PD models do not reflect this. A validated ELISA quantified pembrolizumab levels in 15 samples within 123 days after the administration did show some interesting effects on clearance after 15 days, suggesting drug exposure measurements can be helpful if samples are taken at appropriate times. For example, after day 77, accelerated non-linear clearance observed suggested that the pembrolizumab drug targets were fully saturated at concentrations above 0.6 µg/mL, 43 to 61 times lower than the steady-state trough levels of the currently registered fixed-dose regimens.

Cardiac arrhythmias associated with immune checkpoint inhibitors detected by the FDA adverse event reporting system (FAERS) were investigated by Wang et al.. Specifically, the clinical characteristics of patients reported with ICI-related cardiac arrhythmias were compared between fatal and non-fatal groups, and the time to onset following different ICI regimens was further investigated. Nearly 2000 ICI–associated cardiac arrhythmias were reported, greater in men than women, and more were reported in patients with lung, pleura, thymus, and heart cancers (38.02% of 1957 patients). Interestingly, the spectrum of arrhythmias induced by ICIs differed among therapeutic regimens, but there appeared to be no difference in the onset time between monotherapy and a combination regimen. Moreover, reports of ICI-associated arrhythmias were associated with other concurrent cardiotoxicity, much of which can be explained by the types of cancers patients that with heart disease are co-associated with and the fact that ICIs are currently used (e.g., smoking and lung cancer).

Taken together, this Research Topic provides an overview of *Therapeutic Drug Monitoring and Clinical Toxicology of Anti-Cancer Drugs II* to summarize and confirm that TDM for clinical antineoplastic drugs can better serve patients and improve drug safety.

## Author contributions

All authors listed have made a substantial, direct, and intellectual contribution to the work, and approved it for publication.

